# Supplementary material for: Building consensus on priority areas for Sub-Saharan Africa’s ageing population research: An e-Delphi study protocol
Source: PLoS One. 2024 Apr 11;19(4):e0298541. doi: 10.1371/journal.pone.0298541 (PMC11008814; doi:10.1371/journal.pone.0298541)
Supplement: Attachment — Submitted filename: Response to Reviewers - PONE-D-23-20229.docx [file pone.0298541.s001.docx]

Dear Editor,

Thank you for reviewing our manuscript "Building consensus on priority areas for Sub-Saharan Africa’s ageing population research: an e-Delphi study protocol" and for giving us the opportunity to improve it. All the changes in the revised manuscript were made using "track changes". We have also provided point-by-point response to the reviewer’s comments. We would be very happy, if the revised manuscript will now fulfill the high standards of PLOS ONE for publication. Thank you again for consideration.

Yours sincerely,

Augustine Okoh (For the authors)

**Response to the comments**

*Journal Requirements:*

1. Manuscript has been formatted according to PLOS ONE style requirement

2. We have not completed participant recruitment and the cover letter has been updated to reflect that point.

3. The group (Emerging Researchers and Professionals in Ageing-African Network) members have been listed in the Acknowledgment.

4. We have included an abstract after the title page.

5. The body of this protocol paper contains an introduction section and a material/methods section. The ethics statement is written in the methods section.

6. We have updated the references.

*7. We converted the figure to tif

*Changes in the protocol:*

1. In the background section, particularly in the second paragraph, there is a need to add more and updated references Also evidently state the research gap to comment on how your study is novel in context to low and middle-income countries.

We have provided the reference of a more WHO programme on healthy ageing. regarding the gap, this study intends to probe subject areas and methodologies that would advance inclusion of disadvantaged groups in research priorities for the LMICs.

2. The authors have stated the background of RAND cooperation and Delphi approach in the methodology section along with references while this should be mentioned only in the introduction part.

The statements have been moved to the introduction section.

3. Please specifically explain the category of non-medical caregivers’ stakeholders concerning the areas of their expertise Also, mention the rationale for the exclusion of North African countries in your study.

unlike healthcare professionals, the non-medical caregivers imply individuals providing non-specialized care to an older adult.

4. Please include more clarity on the sampling technique used

First, [researchers] stakeholders will be recruited purposively from a list of the SSA contacts we identified in the preceding phases of this four-part study (XX name blinded for peer review). We will invite the authors from the 544 articles included in our previous reviews (XX ref: blinded for peer review). Subsequently, snowballing method will be used until the desired sample size is reached [18]. We will encourage the stakeholders who were purposively selected to send us the name and email contact of other potential stakeholders who meet our inclusion criteria [14]. Regarding the recruitment of other stakeholders [healthcare practitioners, older adults, and non-medical caregivers], we will contact older people’s homes, faith-based organizations, and geriatric organizations in SSA (e.g., South African Geriatrics Society). We will also contact academic organizations (e.g., Ibadan Longitudinal studies in aging centre, Centre for Ageing Research at the University of Ghana, Health and Aging in Africa: A Longitudinal Study of an INDEPTH Community [HAALSI], etc.), and other groups like HelpAge-Africa.

5. Please use updated references for references # 5, 18, 29 and 38.

**Previous (#5)** Adler NE, Stewart J. Health disparities across the lifespan: Meaning, methods, and mechanisms. Ann N Y Acad Sci. 2010;1186: 5–23. doi:10.1111/J.1749-6632.2009.05337.X 🡪 I will keep this reference because it is still relevant and I did not find another relevant study that discussed the message I wanted to convey.

**Previous (#18 - 19)** Hasson F, Keeney S, McKenna H. Research guidelines for the Delphi survey technique. J Adv Nurs. 2000;32: 1008–1015. 🡪 Niederberger, M., & Spranger, J. (2020). Delphi technique in health sciences: a map. *Frontiers in public health*, *8*, 457.

**Previous (#29 - 33)** Clibbens N, Walters S, Baird W. Delphi research: Issues raised by a pilot study. Nurse Res. 2012;19. doi:10.7748/nr2012.01.19.2.37.c8907 🡪 McPherson, S., Reese, C., & Wendler, M. C. (2018). Methodology update: Delphi studies. *Nursing research*, *67*(5), 404-410.

**Previous (#38 - 42)** Hsieh H-F, Shannon SE. Three Approaches to Qualitative Content Analysis. 2005. doi:10.1177/1049732305276687 🡪 replaced with Kyngäs, H. (2020). Qualitative research and content analysis. *The application of content analysis in nursing science research*, 3-11.

*6. statement regarding the exclusion of North Africa added to the description of the research context

Unlike the Arab-Asiatic North Africa, SSA is mostly inhabited by Black people and the categorization is based on race science and assume a homogeneity of Black experience (Henn et al., 2012). Research priorities and interests tend to differ between North Africa and SSA because of perceived differences in challenges and dynamics as well as distinct historical background, political dynamics, and economic structures in these regions (Abebe & Ofosu-Kusi, 2016; Odigbo et al., 2023).
